# Supplementary material for: Orexin neurons play contrasting roles in itch and pain neural processing via projecting to the periaqueductal gray
Source: Commun Biol. 2024 Mar 8;7:290. doi: 10.1038/s42003-024-05997-x (PMC10923787; doi:10.1038/s42003-024-05997-x)
Supplement: Supplementary file 2 — Supplementary Information [file 42003_2024_5997_MOESM2_ESM.pdf]

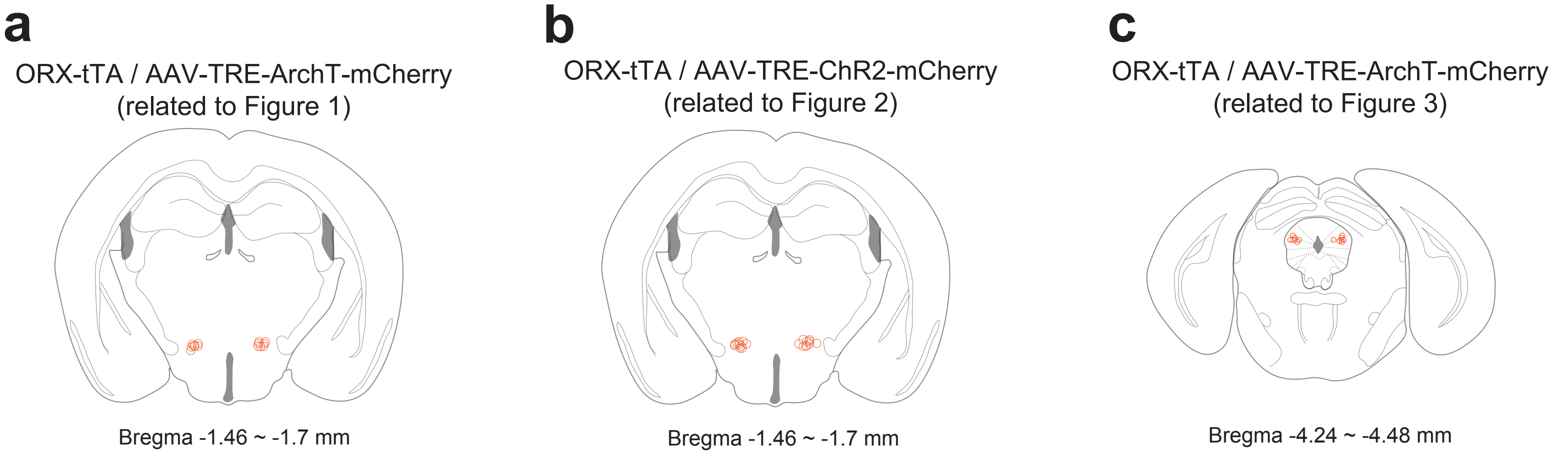

Supplementary Figure 1. Histological confirmation of optical fiber position, related to Figures 1-3.

(a) Fiber placements for optogenetic inhibition of ORX neurons in LH (n=7).
The positions of the rostrocaudal axis were -1.46 ~ -1.7 mm from Bregma.

(b) Fiber placements for optogenetic activation of ORX neurons in LH (n=10).
The positions of the rostrocaudal axis were -1.46 ~ -1.7 mm from Bregma.

(c) Fiber placements for optogenetic terminal inhibition of ORX neurons in lPAG/vlPAG (n=8).
The positions of rostrocaudal axis were -4.24 ~ -4.48 mm from Bregma.

The red circle indicates each optical fiber tip.

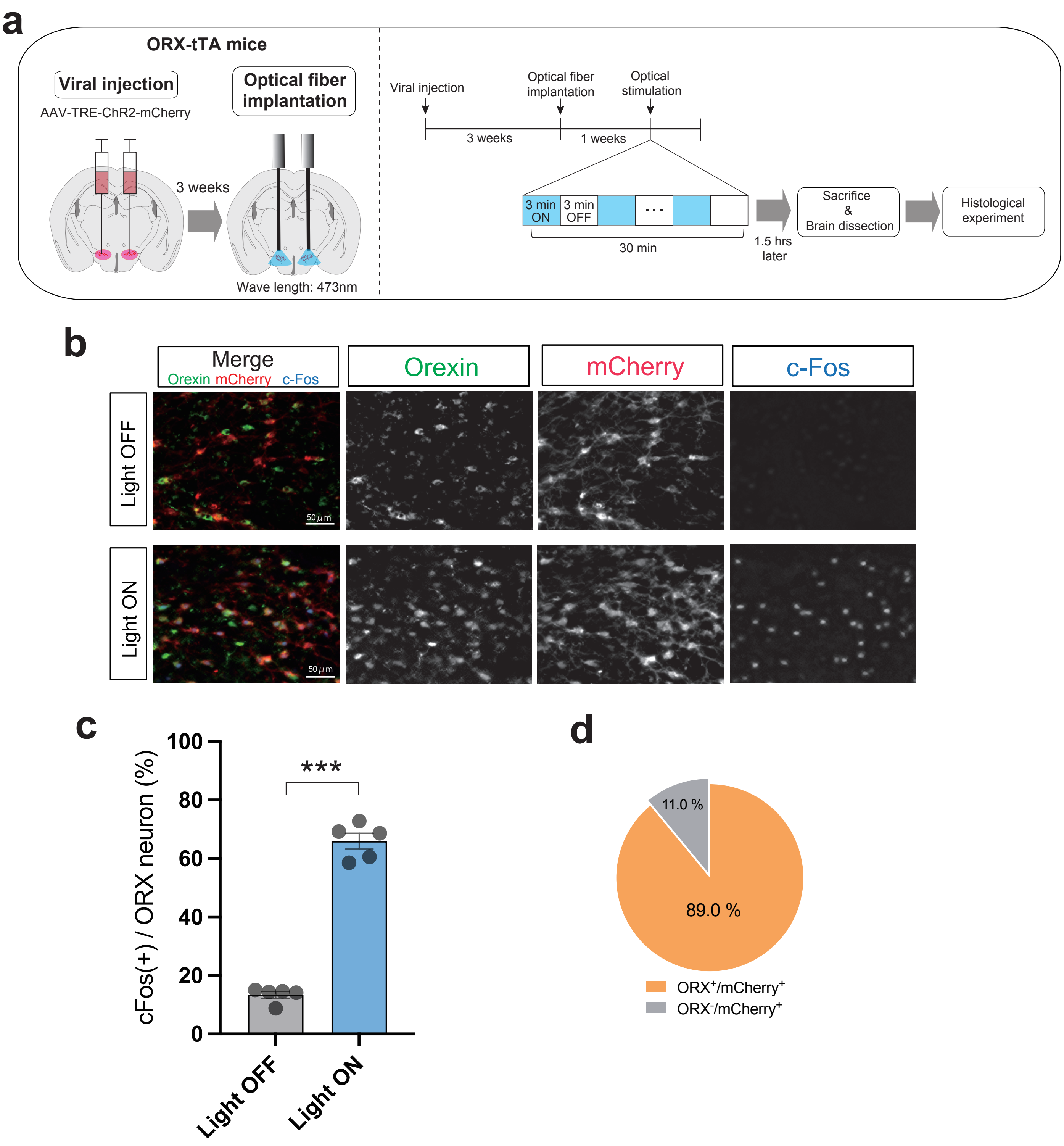

Supplementary Figure 2. The c-Fos expression level of orexin neurons by channelrhodopsin-2 (ChR2)-induced optical stimulation, related to Figure 2.

(a) Left, schematic showing the injection of the AAV-TRE-ChR2-mCherry into the LH of ORX-tTA mice and implantation of the optical fiber. Right, the timeline of the experiments.

(b) The representative pictures of c-Fos expression (blue) in orexin neurons (green) expressing ChR2-mCherry (red) under Light-ON or Light-OFF conditions. Scale bars, 50 μm.

(c) The quantification of c-Fos expression showed a significant increase in c-Fos-positive cells in orexin neurons (%) after the optical stimulation (Light ON) compared with the Light OFF control group. n = 5 for each group.

(d) The quantification (inset pie chart) shows the co-expression of ChR2-mCherry with orexin in LH of ORX-tTA mice used in the experiment (n=3 sections per animal from 10 animals).

The data represent the mean ± SEM. ***$p < 0.001$; unpaired t-test.

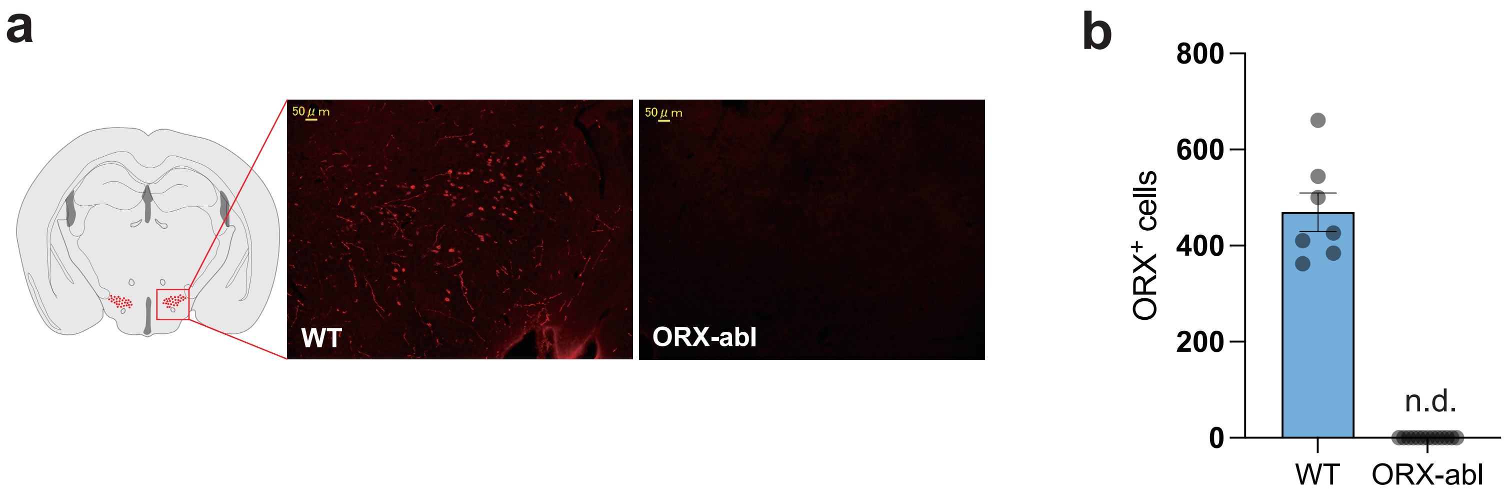

Supplementary Figure 3. Histological confirmation of the ablation of orexin neurons in ORX-abl mice, related to Figure 4.

(a) Histological confirmation of the ablation of orexin neurons in representative ORX-abl mice compared to WT control mice. Scale bars, 50 μm.

(b) The quantification shows no orexin-immunopositive cells were detected (n.d.) in ORX-abl mice (n=13) compared to WT (n=7). The numbers of ORX-positive cells were counted from every 4th section in an animal (six sections per mouse, 40μm thickness for each section), as orexin neurons were distributed over a rostrocaudal distance of ~1mm centered on the LH coordinates. Then, the total number of ORX-positive cells was calculated from six sections per mouse and plotted.

The data represent the mean ± SEM.
